# Genetic diversity and genome-wide association study of 13 agronomic traits in 977 *Beta vulgaris* L. germplasms

**DOI:** 10.1186/s12864-023-09522-y

**Published:** 2023-07-24

**Authors:** Dali Liu, Wenbo Tan, Hao Wang, Wangsheng Li, Jingjing Fu, Jiajia Li, Yuanhang Zhou, Ming Lin, Wang Xing

**Affiliations:** 1 National Beet Medium-term Gene Bank, Heilongjiang University, Harbin, 150080 P. R. China; 2 Key Laboratory of Sugar Beet Genetics and Breeding, College of Advanced Agriculture and Ecological Environment, Heilongjiang University, Harbin, 150080 P. R. China; 3 Xinjiang Academy of Agricultural Sciences, Urumqi, 830091 P. R. China

**Keywords:** Sugar beet (*Beta vulgaris* L.), Genotyping-by-sequencing (GBS), Genome-wide association study (GWAS), Descriptive traits, Candidate genes

## Abstract

**Background:**

Sugar beet (*Beta vulgaris* L.) is an economically essential sugar crop worldwide. Its agronomic traits are highly diverse and phenotypically plastic, influencing taproot yield and quality. The National Beet Medium-term Gene Bank in China maintains more than 1700 beet germplasms with diverse countries of origin. However, it lacks detailed genetic background associated with morphological variability and diversity.

**Results:**

Here, a comprehensive genome-wide association study (GWAS) of 13 agronomic traits was conducted in a panel of 977 sugar beet accessions. Almost all phenotypic traits exhibited wide genetic diversity and high coefficient of variation (CV). A total of 170,750 high-quality single-nucleotide polymorphisms (SNPs) were obtained using the genotyping-by-sequencing (GBS). Neighbour-joining phylogenetic analysis, principal component analysis, population structure and kinship showed no obvious relationships among these genotypes based on subgroups or regional sources. GWAS was carried out using a mixed linear model, and 159 significant associations were detected for these traits. Within the 25 kb linkage disequilibrium decay of the associated markers, NRT1/PTR FAMILY 6.3 (*BVRB_5g097760*); nudix hydrolase 15 (*BVRB_8g182070*) and TRANSPORT INHIBITOR RESPONSE 1 (*BVRB_8g181550*); transcription factor MYB77 (*BVRB_2g023500*); and ethylene-responsive transcription factor ERF014 (*BVRB_1g000090*) were predicted to be strongly associated with the taproot traits of root groove depth (RGD); root shape (RS); crown size (CS); and flesh colour (FC), respectively. For the aboveground traits, UDP-glycosyltransferase 79B6 (*BVRB_9g223780*) and NAC domain-containing protein 7 (*BVRB_5g097990*); F-box protein At1g10780 (*BVRB_6g140760*); phosphate transporter PHO1 (*BVRB_3g048660*); F-box protein CPR1 (*BVRB_8g181140*); and transcription factor MYB77 (*BVRB_2g023500*) and alcohol acyltransferase 9 (*BVRB_2g023460*) might be associated with the hypocotyl colour (HC); plant type (PT); petiole length (PL); cotyledon size (C); and fascicled leaf type (FLT) of sugar beet, respectively. AP-2 complex subunit mu (*BVRB_5g106130*), trihelix transcription factor ASIL2 (*BVRB_2g041790*) and late embryogenesis abundant protein 18 (*BVRB_5g106150*) might be involved in pollen quantity (PQ) variation. The candidate genes extensively participated in hormone response, nitrogen and phosphorus transportation, secondary metabolism, fertilization and embryo maturation.

**Conclusions:**

The genetic basis of agronomical traits is complicated in heterozygous diploid sugar beet. The putative valuable genes found in this study will help further elucidate the molecular mechanism of each phenotypic trait for beet breeding.

**Supplementary Information:**

The online version contains supplementary material available at 10.1186/s12864-023-09522-y.

## Background

Within the *Amaranthaceae* family, sugar beet (*Beta vulgaris* L.) is native to the western and southern coasts of Europe and is mostly biennial, with the exception of some wild beets without swollen roots [1]. The taproot of sugar beet is the primary source of sucrose, and the annual beet root output worldwide can reach 253 million tons, which provides approximately 30% of the world’s gross requirements for white sugar [2]. In addition to being used for sugar production, beet can also be consumed as a food, fed to livestock or even transformed into ethanol [3]. Due to its economic importance and broad adaptability, sugar beet is widely planted worldwide, including in northern China. The National Beet Medium-term Gene Bank in China (Harbin, Heilongjiang Province) focuses on the collection, propagation, preservation, innovation and utilization of sugar beet germplasm resources as natural allelic variants and accelerating the breeding of sugar beet with high yield, quality and disease resistance by genetic improvement using excellent germplasm hybridization. The abundant germplasm resources here provide a broad genetic basis for sugar beet genetic research.

The agronomic characteristics of sugar beet, such as granularity, leaf bush type, root size, and root groove depth, are important for agricultural practices, and they are also of concern to growers and sugar manufacturers. Further genetic improvement of sugar beet will require an understanding of the genomic regions and genes that govern these specific traits. Sugar beet is a self-incompatible, typically outcrossing crop, and the total genetic diversity of sugar beet along with other *Beta* species, including other cultivated beet crops and their wild relatives, is quite high [4]. In addition to genetic variation, the performance of sugar beet is also influenced by various environmental and agronomic factors that ultimately determine the economic yield [5]. Therefore, sugar beet serves as an excellent model crop for studying the genetic architecture of agronomic traits related to yield or physiology [6] due to its varying complexity in seed, leaf, root, pollen and other growth- and development-related phenotypes.

Understanding the genetic basis of phenotypic traits is a major challenge in crops, and sugar beet is no exception. Most heritable components of agronomic performance, which are highly correlated with morphological values, can be assayed with genetics- and genomics-based methods. By analysing phenotypic and genotyping data, the genetic architecture of important agronomic and physiological traits such as α-amino nitrogen or sugar content in sugar beet was uncovered by detecting main-effect quantitative trait loci (QTLs) [6], and promising candidate loci correlated with genotypic variance were validated for use in further breeding. Another study identified a total of 32 QTLs for sugar yield-related traits, and QTL mapping and chromosomal marker distribution data were used to screen 3690 candidate genes, including 191 root length, 918 root perimeter, 409 root weight, and 2172 sugar content genes [7]. Sugar beet reference genomes [8], assemblies [9] and sequencing strategies [10] increase researchers’ ability to rapidly and accurately characterize genome variation within diverse beet germplasms. By using a modified method of mapping-by-sequencing (MBS), Capistrano-Gossmann et al. identified the sugar beet resistance gene *Rz2* in a crop wild relative (CWR) population of < 200 wild beets [11]. Twenty-four nonbolting and 15 bolting beets of the L14 line were sequenced by restriction site-associated DNA sequencing (RAD-seq), and the single-nucleotide polymorphism (SNP) markers SNP_36780842 and SNP_48607347 were found to be associated with low bolting tendency by a genome-wide association study (GWAS) [12]. Two extreme phenotypes were used for bulk segregant analysis by RAD-seq, and the SNP10139 sequence was mapped to the *B. vulgaris* peptide transporter (*PTR*) gene, a carrier that influences root elongation [13]. Using pooled whole-genome sequencing (WGS) of the outcrossing sugar beet population EL57 (PI 663,212), which displays rhizoctonia resistance, a series of candidate genes were found to possibly function in plant disease resistance [14]. By weighted gene coexpression network analysis (WGCNA) of differentially expressed genes (DEGs) identified by RNA-seq, Cui et al. found a total of 41 hub genes related to salt stress resistance in the beet cultivar O68 [15].

Next-generation sequencing (NGS) technology provides an effective way to obtain genetic information on sugar beet, and many genes associated with important agronomic traits can be exploited, which could then be used to further improve breeding. Li et al. obtained a high-quality, chromosome-level genome assembly for the pure line IMA1 [16]. These genomic resources in sugar beet have enabled GWAS for the identification of 10 disease-resistance genes associated with three important beet diseases, 5 genes associated with sugar yield per hectare and 9 highly expressed genes associated with pollen fertility in sugar beet. Although significant progress has been made via different approaches in recent years, genetic variability and key genes associated with essential agronomical traits related to sugar beet phenotypes remain unexplored. Genotyping-by-sequencing (GBS) is one of the most promising approaches for genomic characterization [17]. By searching for significant genotype and phenotype associations using SNPs, the GBS-GWAS approach can be successfully applied to explore the genetic architecture and associated genes of agronomic traits in different germplasms. To determine phenotype–genotype associations, 977 accessions from 21 countries in the National Beet Medium-term Gene Bank of China were characterized at both the genetic and phenotypic levels by extensively studying their population structure, phylogenetic relationships, patterns of linkage disequilibrium (LD) and phenotypic and genetic diversity, and our objectives were to (1) evaluate the genetic diversity and elucidate the phylogenetic relationships of 977 sugar beet accessions, (2) conduct a GWAS for systematic identification of associated genomic regions for 13 descriptive traits, (3) identify putative candidate genes related to these agronomic traits, and (4) provide valuable insight into the genetic architecture of sugar beet agronomic traits and genetic resources for accelerating sugar beet genomic breeding.

## Results

### Phenotypic trait evaluation

A total of 977 accessions were evaluated under field conditions, and the 13 descriptive traits related to sugar beet phenotypes were screened for assessment. These germplasms displayed all phenotypic variations of these 13 traits (Table 1). There were three plant types (PTs), and the differences in proportions among them were relatively small (27.3-40.9%). The pollen quantity (PQ) of sugar beet was mostly medium (38.5%) or high (48.3%), and 62.0% of hypocotyls were of a mixed red and green colour (hypocotyl colour, HC). Among the leaf traits, the petiole length (PL) and width (PW), cotyledon size (C) and fascicled leaf type (FLT) had 3 types, and most accessions showed a semicrawl phenotype (87.7%) with a medium PL (49.8%) and PW (59.0%) and large cotyledon (61.7%); a few petioles were narrow (9.6%) and short (17.4%). The distribution of phenotypic traits of taproots was different. The root shape (RS) and root skin (SR) were mainly conical (69.8%) and very smooth (67.1%), respectively. The distribution of crown size (CS) was relatively uniform, and only a few taproots had inconspicuous root grooves (RGD). Among all genotypes, 88.1% displayed a white taproot flesh colour (FC), and pink and red accounted for only 0.4% and 1.4% of the accessions, respectively. Growth vigour (GV) was divided into five levels and ranged from 5.6% (very weak) to 34.0% (vigorous).

**Table 1:** Distribution frequency, coefficient of variation and genetic diversity of the 13 descriptive traits pollen quantity (PQ), plant type (PT), hypocotyl colour (HC), cotyledon size (C), petiole width (PW), petiole length (PL), fascicled leaf type (FLT), root shape (RS), crown size (CS), root groove depth (RGD), skin roughness (SR), flesh colour (FC) and growth vigour (GV) in 977 sugar beet germplasms

Traits	Characteristic description (proportion of distribution, %)					CV (%)	Index of genetic diversity/H’
	1	2	3	4	5		
PQ	Little (13.2)	Medium (38.5)	Much (48.3)			29.97	0.986
PT	Single stem (27.3)	Many stems (40.9)	Mixture (31.8)			37.55	1.084
HC	Green (9.6)	Red (28.4)	Mix (62.0)			26.34	0.879
C	Small (11.1)	Medium (27.2)	Large (61.7)			27.45	0.896
PW	Narrow (9.6)	Medium (59.0)	Broad (31.4)			27.16	0.900
PL	Short (17.4)	Medium (49.8)	Long (32.8)			32.10	1.017
FLT	Erect (8.2)	Semicrawl (87.7)	Crawl (4.1)			17.78	0.451
RS	Conical (69.8)	Cuneiform (17.2)	Spindle (12.2)	Regular (0.8)		51.08	0.849
CS	Small (40.0)	Medium (23.0)	Large (37.0)			44.53	1.073
RGD	Not obvious (1.6)	Shallow (63.9)	Deep (34.5)			22.07	0.718
SR	Very smooth (67.1)	Smoother (18.1)	Very rough (14.8)			50.03	0.859
FC	White (88.1)	Light yellow (10.1)	Pink (0.4)	Red (1.4)		40.46	0.424
GV	Very vigorous (20.2)	Vigorous (34.0)	Medium (30.7)	Weak (9.5)	Very weak (5.6)	44.12	1.438

The genetic diversity and coefficient of variation (CV) of these phenotypic traits were different (Table 1). The investigation and analysis of these descriptive characteristics yielded an average CV and Shannon information index of 34.66% and 0.890, respectively. The Shannon information index (H’) ranged from 0.424 (FC) to 1.438 (GV), and the CVs were between 17.78% (FLT) and 51.08% (RS). Most phenotypic traits showed excellent genetic diversity, especially PT, CS, PL and GV, with a diversity index exceeding 1. RS, skin roughness (SR), CS, GV and FC showed substantial variation (≥ 40%). These results indicated that a few agronomic traits of sugar beet were stable, but most of them had rich variability. Trait variation frequency is used to quantify phenotypic diversity, and the greater the CV is, the higher the richness of the breeding materials.

### Genotyping-by-sequencing of the sugar beet genome and characterization of SNPs

GBS yielded approximately 894.027 Gb in total for the 977 sugar beet accessions (Table S1). The amount of high-quality clean data obtained was 893.989 Gb, with an average of 0.915 Gb per sample. The sequencing quality was high, and the GC distribution was normal. The sugar beet genome size was 566,550,431 bp, and approximately 97.61% of the reads were successfully mapped to the sugar beet reference genome (RefBeet-1.2.2). The average sequencing depth of the genome was 15.17X, and the average coverage was 17.71% (at least one base was covered).

A total of 4,561,550 SNP loci were detected with GATK software. After filtering under the conditions of DP4, MISS0.2 and MAF0.05, 170,750 high-quality SNPs were finally obtained. Among these SNPs, 108,952 were mainly located in intergenic regions. There were 5131 SNPs located in the 1 kb region upstream of the genes. A total of 5882 nonsynonymous mutations were found in exons of the chromosome coding region (Table S2). High-quality GBS-derived SNPs were used for the following population structure analysis and GWAS.

### Genetic relationship and population structure analysis

**Fig. 1:**
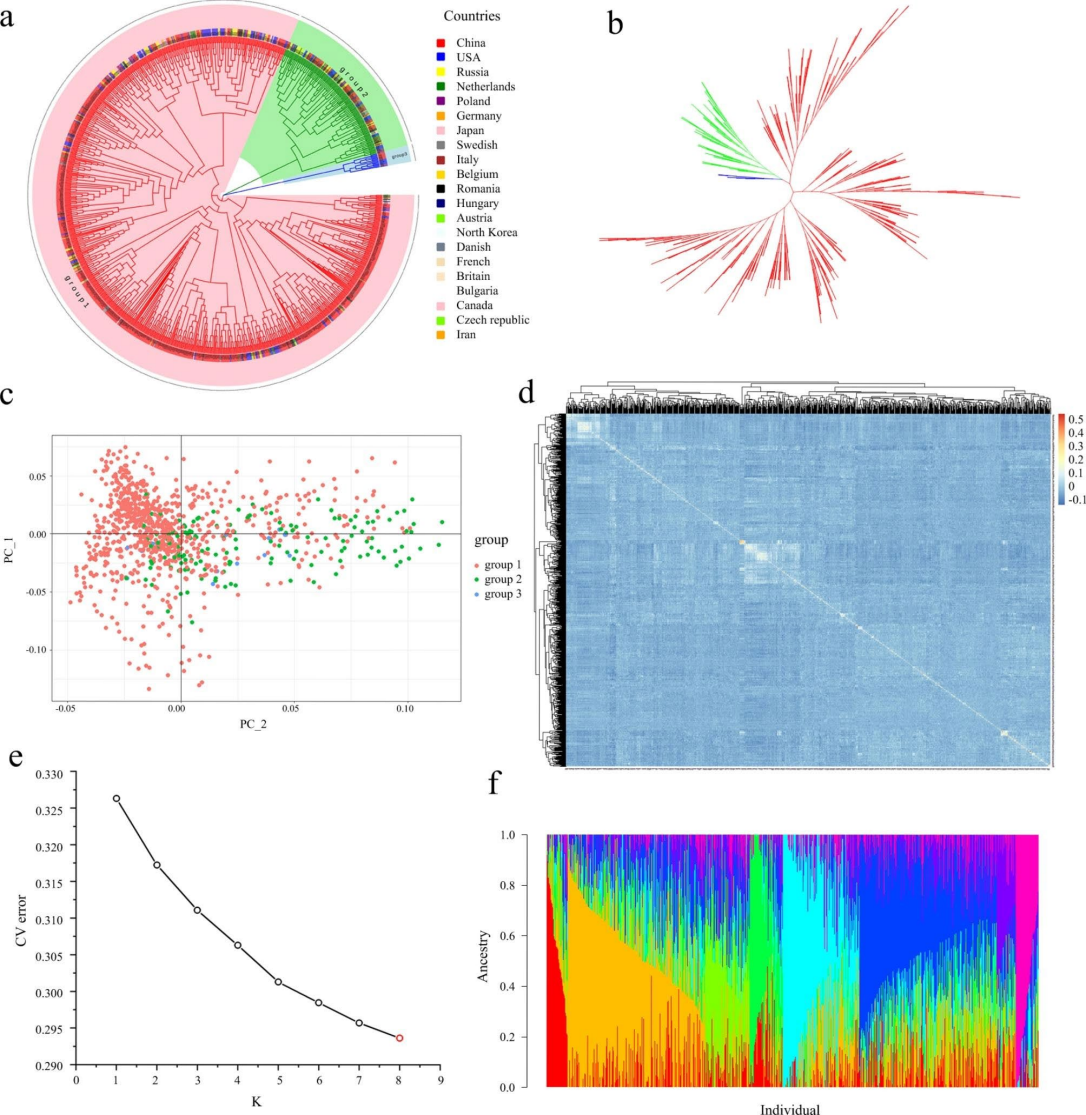
Genetic structure of 977 sugar beets based on the analysis of GBS-derived SNPs **a and b** Rooted and unrooted phylogenetic trees of sugar beet collections generated using the neighbour-joining method. Different colours represent the subpopulations identified **c** Principal component analysis (PCA) differentiating the 977 accessions. Different subgroups are shown in different colours. PC 1 and PC 2 refer to the first and second principal components, respectively **d** Kinship matrix of 977 genotypes based on the TASSEL program **e and f** Population structure of 977 sugar beets at K = 8 as selected by the cross-validation (CV) error value. Each cultivar is represented by a single vertical line, and the same colour represents one cluster

To determine the evolutionary relationships among the 977 sugar beet accessions, a maximum likelihood-based phylogenetic tree was constructed using the NJ method based on the GBS-derived SNP genotypes. As shown in Fig. 1a and b, there were three clusters, and cluster I comprised 83.62% of all materials, most of which came from China; cluster I could be further divided into four major subgroups. The second group contained germplasms from almost all the countries of origin, but group III included materials only from China and the USA. The clustering of all individuals in each population was relatively strong.

Using the nucleotide polymorphisms, we performed principal component analysis (PCA) to quantify the population structure of these 977 panels (Fig. 1c). The principal components showed a continuous distribution without apparent distinct clusters, and the first two principal components explained only 1.905% and 1.464% of the total variance, respectively, indicating that the genotypes did not represent a highly structured population [18]. Marker-based kinship was also estimated throughout all the panels (Fig. 1d), and almost all of the kinship coefficients were below 0.1, indicating that most accessions had a weak genetic relationship with the other accessions [19], which might be attributed to the extensive exchange of sugar beet germplasms.

ADMIXTURE software was used to calculate the genetic components of each panel (Fig. 1e and f). A K value of 8 had the lowest cross-validation (CV) error and thus was considered the number of subpopulations. These genotypes did not show extremely strong population structure.

### Genome-wide association analysis

**Fig. 2:**
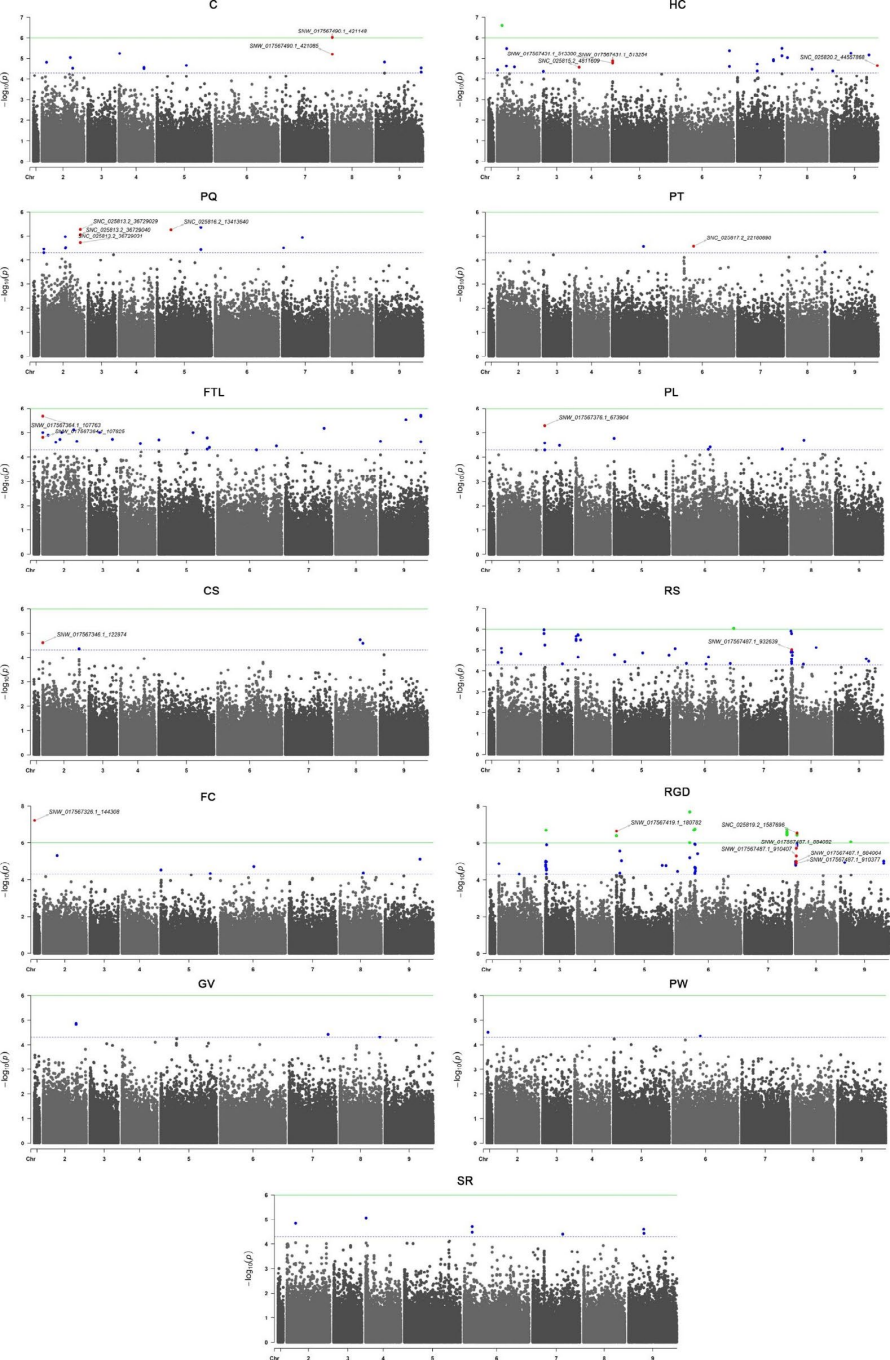
Manhattan plots for the descriptive traits in the FULL panel analysed by genome-wide association study (GWAS) using a mixed linear model. The y-axis shows − log_10_(*p*) for different traits, and each dot represents a SNP. PQ, pollen quantity; PT, plant type; HC, hypocotyl colour; C, cotyledon size; PW, petiole width; PL, petiole length; FLT, fascicled leaf type; RS, root shape; CS, crown size; RGD, root groove depth; SR, skin roughness; FC, flesh colour; GV, growth vigour

Using a linear mixed model with correction based on kinship bias, we performed a GWAS for the 13 descriptive traits across 977 panels. The false-positive rate of the GWAS was controlled adequately according to quantile‒quantile (Q-Q) plots, which showed that the expected value (red line) was roughly equal to the observed value (red dot) after controlling for Q and K (Fig. S1). A total of 159 significantly associated SNPs with (- log_10_ (*p*)) ≥ 4.5 were detected on all nine chromosomes (Fig. 2; Table S3). Among these associations, the abundant SNPs were mainly located on chromosomes 2 (30) and 8 (29), there was only one significant association with PW and FC located on chromosome 1, and approximately 10 to 20 SNP loci were distributed on the other chromosomes. Specifically, the largest number of the 52 significant SNPs was identified for RGD, which were present on all chromosomes except for 1 and 4, and the distance between the significant markers, such as SNC_025819.2_1587696, SNC_025819.2_1587724 and SNC_025819.2_1587813, was less than 100 bp. In addition, 29, 22, 18, 11 and 9 significant SNPs were associated with RS, FLT, HC, C and PQ, respectively. The other traits were associated with no more than four SNPs, with a *P* threshold ≥ 4.5. The screened SNPs linked with different traits exhibited different distribution characteristics on these chromosomes. SNW_017567365.1_890035 and SNW_017567495.1_345173 were identified to be related to both RS and RGD.

### Candidate gene identification

**Fig. 3:**
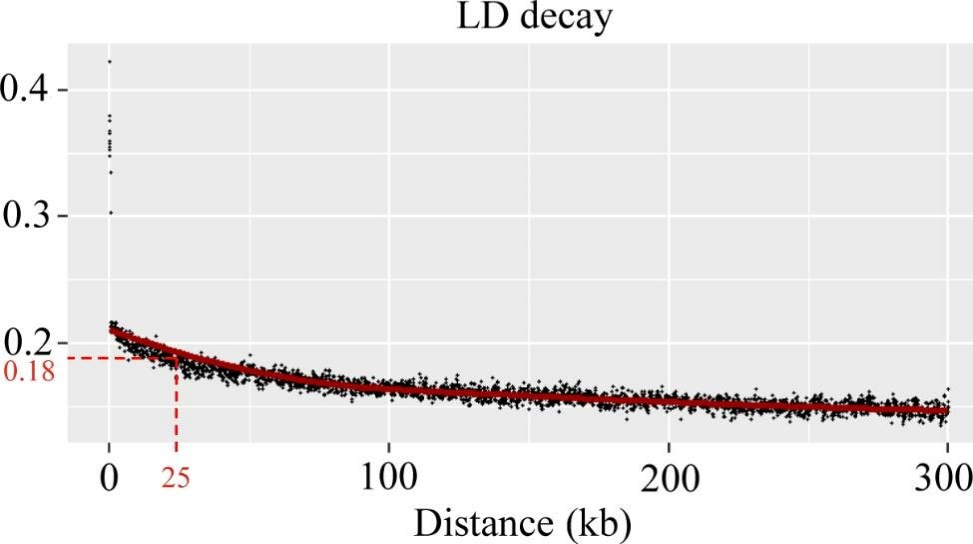
Chromosome-wide linkage disequilibrium (LD) decay estimated from SNPs of 977 sugar beet genotypes

The level of LD can determine the density of markers required for association analysis and the accuracy of association analysis to a certain extent. To evaluate the positioning accuracy of association analysis, the estimate of *r*^*2*^ for all pairs of linked SNP loci was used to assess the extent of LD decay in this study. As expected, the *r*^*2*^ value declined with increasing physical distance between markers. The average *r*^*2*^ for the whole genome decreased to half (0.18) of its maximum value at a 25 kb distance, which resulted in the inclusion of fewer and more accurate candidate genes within an LD block (Fig. 3).

**Fig. 4:**
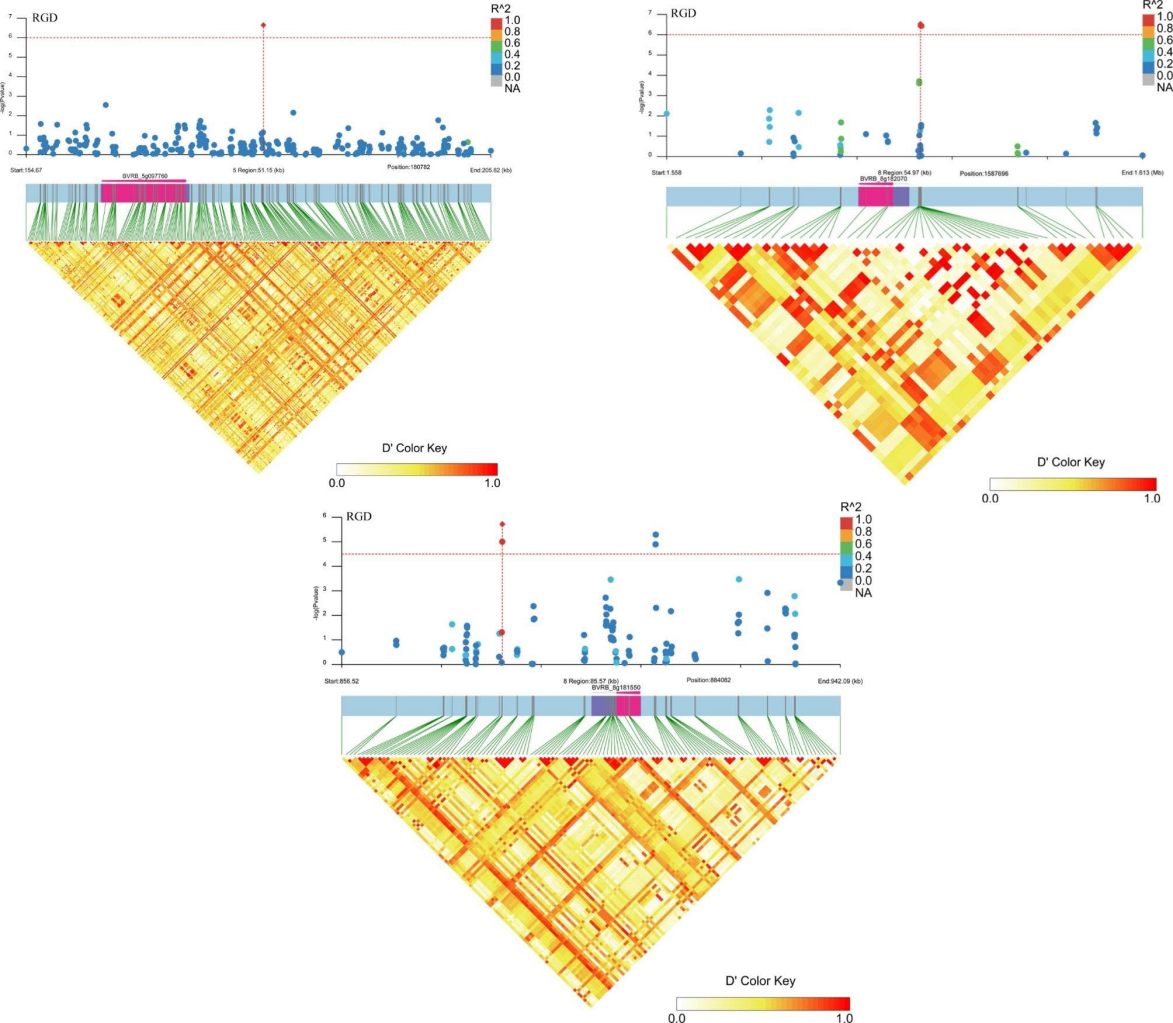
Manhattan plot and LD heatmap of the candidate genes for RGD. The orange vertical dotted line indicates the position of significantly associated SNPs, and the orange horizontal line indicates -log_10_*p*

**Fig. 5:**
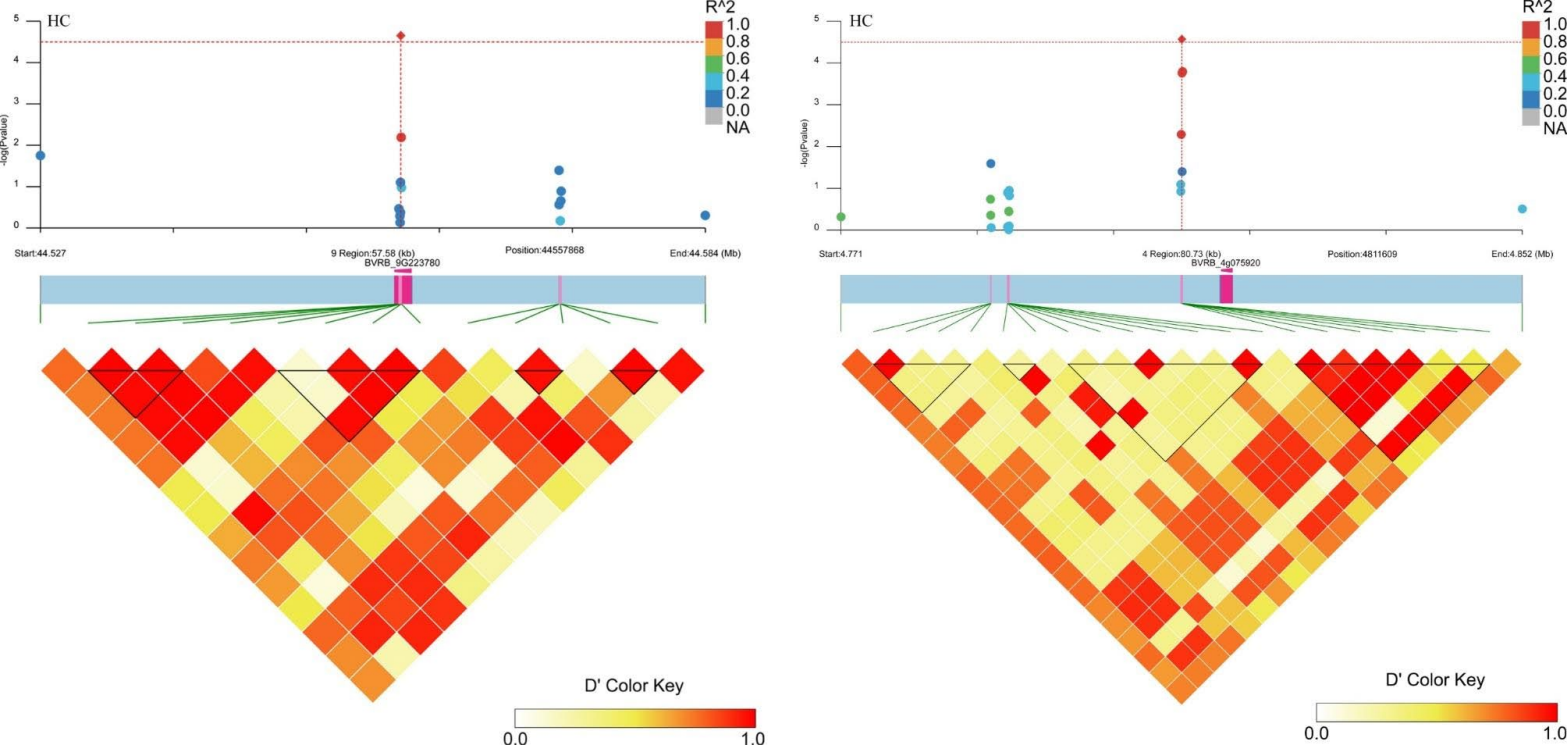
Manhattan plot and LD heatmap of the candidate genes for HC. The orange vertical dotted line indicates the position of significantly associated SNPs, and the orange horizontal line indicates -log_10_*p*

**Fig. 6:**
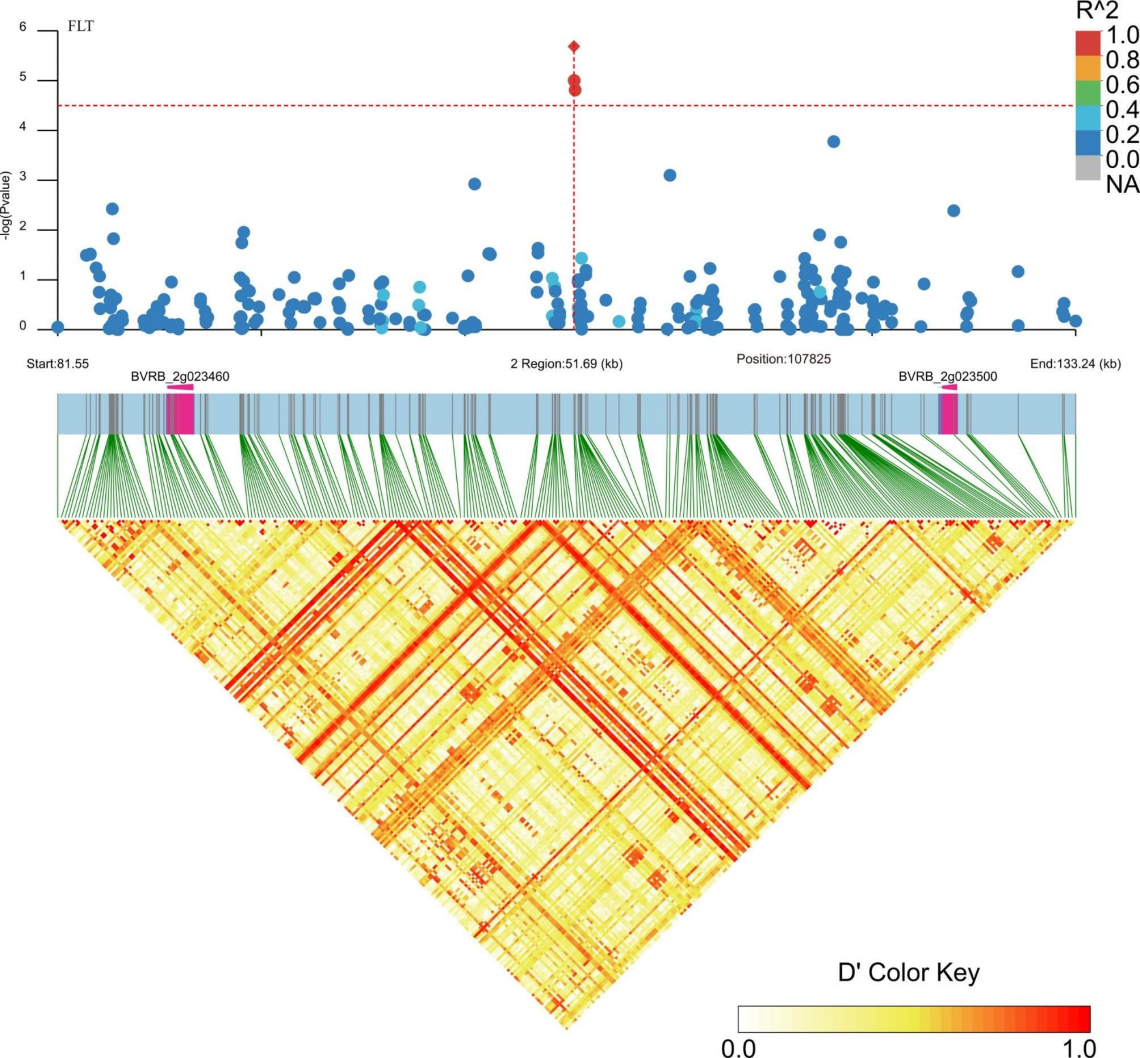
Manhattan plot and LD heatmap of the candidate genes for FLT. The orange vertical dotted line indicates the position of significantly associated SNPs, and the orange horizontal line indicates -log_10_*p*

**Fig. 7:**
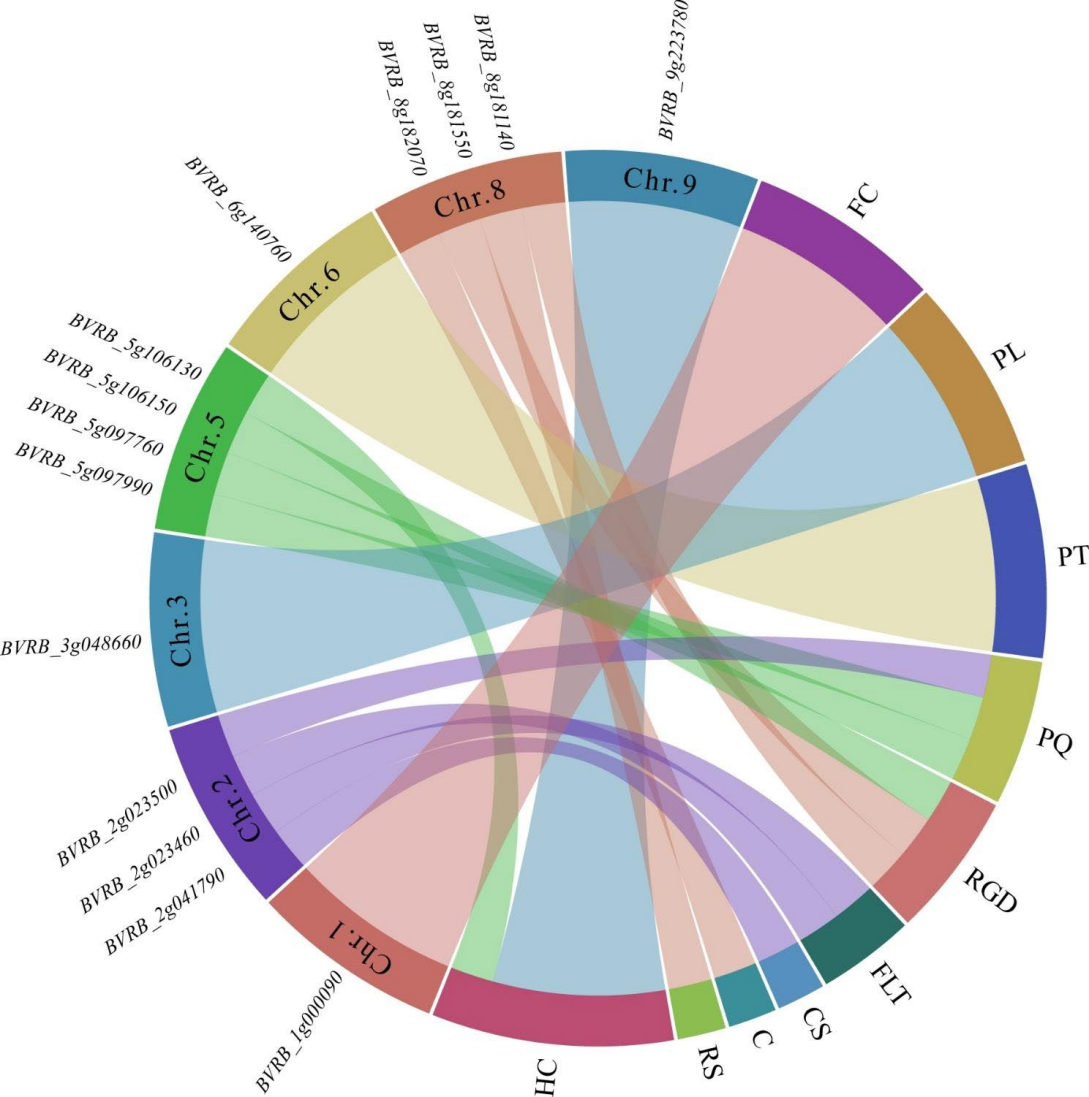
The most important genes associated with RGD, CS, FC, RS, HC, C, PT, PL, PQ and FLT distributed over sugar beet chromosomes

These analyses have allowed the identification of known and novel genes for these descriptive traits. To assess the putative candidate genes, triangle plots for pairwise LD involving significant markers were created for the taproot, leaf, and other plant growth and development agronomic traits (Figs. 4, 5, 6 and 7; Figs. S2-S8; Table S4). The selected genes seemed to be interesting and might play a role in a particular trait. For example, for RGD, *BVRB_5g097760* encoding protein NRT1/PTR FAMILY 6.3 was located in the upstream region of SNW_017567419.1_180782 on chromosome 5; on chromosome 8, putative nudix hydrolase 15 (*BVRB_8g182070*) was found close to SNC_025819.2_1587696, and TRANSPORT INHIBITOR RESPONSE 1 (*BVRB_8g181550*) was present in the effective region of four different markers, and this gene was also associated with RS (Fig. 4; Fig. S2; Table S4). For C, a particular gene encoding the F-box protein CPR1 (*BVRB_8g181140*) was identified downstream of SNW_017567490.1_421148 and SNW_017567490.1_421085 on chromosome 8 (Fig. S3; Table S4). Two genes, *BVRB_9g223780* and *BVRB_4g075920*, belonging to the UDP-glycosyltransferase family were associated with HC (Fig. 5; Table S4); among them, 84B2 contained the SNC_025820.2_44557868 mutation on chromosome 9, while 79B6 was located downstream of SNC_025815.2_4811609. Candidate genes (*BVRB_5g106130*, *BVRB_2g041790* and *BVRB_5g106150*) for PQ were predicted to be AP-2 complex subunit mu, trihelix transcription factor ASIL2 and late embryogenesis abundant protein 18 present in specific regions on chromosomes 2 and 5 (Fig. S4; Table S4). For CS, the *transcription factor MYB77* (*BVRB_2g023500*) downstream of SNW_017567346.1_122974 was discovered on chromosome 2 (Fig. S5; Table S4). The ethylene-responsive transcription factor ERF014 (*BVRB_1g000090*) was found in the linked region of SNW_017567326.1_144308 associated with FC (Fig. S6; Table S4). F-box protein (*BVRB_6g140760*) might be indirectly involved in the variation in PT via the mutation of SNC_025817.2_22180890 on chromosome 6 (Fig. S7; Table S4). Several genes were identified for FLT, including the transcription factor MYB77 (*BVRB_2g023500*) and alcohol acyltransferase 9 (*BVRB_2g023460*) on chromosome 2, with the former also detected for CS (Fig. 6; Fig. S5; Table S4). The SNW_017567376.1_673904 LD region harbours 6 genes, including *BVRB_3g048660*, which encodes the phosphate transporter PHO1 and might be associated with PL (Fig. S8; Table S4). Candidate genes linked with significant SNP loci were also detected for the traits GV and PW but not for SR (Table S4). Most of the genes could be annotated to a protein that is responsible for developmental and physiological processes. However, there were still some hypothetical or uncharacterized candidate genes that might fulfil their role in the formation and variation of these traits and need to be further explored.

## Discussion

Phenotypic variants of most agronomic traits of sugar beet are qualitatively or quantitatively inherited and controlled by multiple genes or QTLs, and they comprehensively affect the growth and development and even the sugar production and processing of beet. Agricultural practices promote the need for developed genotypes with specific agronomic characteristics. In sugar beet, traditional selective breeding is based on traits of morphology, physiology and chemistry [20]. Currently, molecular or biological breeding requires genetic and genomic backgrounds that incorporate both genotype and phenotype. GWASs are a powerful tool for detecting high-density SNPs, identifying genomic loci or genes associated with agronomic traits in crop species, and determining the genetic architecture of complex traits in large germplasm sets [21], which are critical for effective manipulation in crop breeding. GBS facilitates genetic characterization, GWASs, linkage analysis and genomic mapping based on SNPs. It has been demonstrated that by using a panel of unrelated diverse germplasms, candidate gene identification can be significantly improved compared with that for biparental population linkage mapping [18]. Hence, in this study, 977 genotypes of sugar beet collected from 21 countries were included in a GBS-GWAS analysis system to obtain a comprehensive understanding of the genetic associations of approximately 13 descriptive traits.

### Abundant phenotypic diversity and variation in 977 sugar beet genotypes

The diverse traits of leaves, taproots, pollen and even seedlings are important features of the phenotypic diversity of sugar beet. For instance, plant vigour in combination with other seedling traits, such as PT, has significant effects on sugar yield [22]. We used the 977-genotype natural population (with rich genetic variation) preserved in the National Beet Medium-term Gene Bank as the research object to investigate the gene sites in the sugar beet genome that control the target traits. The large amount of variation and phenotypic diversity in these 13 descriptive traits observed among panels indicated abundant genetic diversity among the genotypes. According to the general phenotype or specific proportion of phenotypic data for each tested germplasm and the three controls, the final results were objectively reflected by assigning values to descriptive traits. The Shannon information index and the CV of beet germplasms showed the highest diversity and higher variation in GV (1.438 and 44.12%; Table 1). GV reflects the strength and degree of seedling growth potential, and it is affected by multiple factors, such as cotyledon size and even hypocotyl colour in sugar beet. The taproot traits RS, SR, FC and CS exhibited higher variation than the leaf and other traits (Table 1). Taproot performance was one of the most important agronomic traits in the history of evolution and domestication of sugar beet, and these phenotypic variations accumulated through recombination and selection of genetic sites during hybridization. Similarly, CS remained highly diverse, which might be inherited with and controlled by the same mutations as RGD and SR (Table 1). Root morphological traits are associated with processing quality and continue to influence harvest operations and factory procedures [23]. A lower Shannon information index was found for FC and FLT, and the latter had the lowest CV value (Table 1), indicating that their inheritance was relatively stable. In general, sugar beet is a typical outcrossing crop due to its self-incompatibility [4], and the genetic diversity of these agronomic traits in 977 genotypes was shown to be rich and to cover wide genetic variation. The results indicated that their phenotypic variation was mainly controlled by genetic information and is suitable for marker‒trait association studies.

### Effective population structure analysis enabled by high-quality GBS data

In this study, 977 sugar beet germplasms were sequenced with an average depth of 15.17X by the GBS method (Table S1). The average coverage was 17.71% (covering at least one base), and the coverage of at least four bases was 8.86%. A total of 170,750 SNPs were obtained through screening and filtering, and only approximately 8.0% of them were located in a gene on average. Nonsynonymous mutations that can change the amino acid coding sequence accounted for 43.3% of the genic regions (Table S2), which means that some SNPs on the chromosome may not change the gene sequence and gene function, and only a few SNP mutations play a key role in the evolution process, which should be considered in the analysis of genotype–phenotype associations.

Before analysing genotype–phenotype associations, population stratification analysis must be carried out to avoid false-positive associations. We used a phylogenetic tree, PCA, population structure and a genetic relationship matrix to comprehensively analyse the population structure of 977 genotypes. According to the genotypic data, there were three groups among the 977 accessions, which could be subdivided into six subgroups overall (Fig. 1a and b). Clustering based on the NJ method showed that their genetic distance was not necessarily related to their geographical origin, indicating that the exchange of sugar beet breeding resources was relatively frequent. Sugar beet originated in the Mediterranean and spread globally in a relatively short time, in particular, less than 120 years after it was introduced to China. Therefore, beet germplasm from various sources has not been significantly differentiated due to geographical isolation. Additionally, the majority of sugar beet accessions clustered together with wild beets from many different countries, so it was not possible to assign a single geographic origin to sugar beet [24]. PCA result visualization (Fig. 1c) showed that the germplasm materials from the same clusters were scattered, indicating no obvious close correlation between group aggregation and geographical source. Based on the CV error, the tested sugar beet germplasms were divided into 8 subgroups by population structure analysis (Fig. 1e and f), some of which contained multiple genetic backgrounds, also implying that there was a certain degree of gene exchange between the subgroups. The population structure and genetic relatedness between accessions (Fig. 1d) affect the accuracy of genetic mapping, and the structure matrix is critical for the elimination of spurious marker‒trait associations in GWAS populations [25]. The results of the population structure and relationship analysis showed population stratification and weak relationships between individuals among the 977 experimental materials.

### GWASs reveal putative genes associated with the variation in agronomic traits of sugar beet

To screen and identify excellent sugar beet germplasms, the main agronomic characters are described, and the classification and evaluation criteria of sugar beet are specified in detail [26]. In this study, based on phenotypic data and SNP information at the whole-genome level of 977 beet genotypes, association analysis was carried out to identify the genomic regions significantly associated with the target traits. During GWAS analysis, individual kinship and population stratification are the main factors causing false associations [27]. Therefore, the mixed linear model (MLM) was introduced to simultaneously correct the calculations and screen out potential candidate SNPs on the basis of the significance of association (*P* value) [28]. According to the Q-Q results, the distribution of observed *P* values was closest to the distribution of expected *P* values (Fig. S1), indicating that the results of GWAS analysis performed using the MLM (Q + K) model in this study were more reliable [29]. There were few markers exhibited on chromosome 1 (Fig. 2). It might be due to low quality of sequencing reads on chromosome 1, which were filtered by conditions of DP ≥ 4, missing data ≤ 80% and minor allele frequency (MAF) ≥ 0.05. Besides, Bonferroni correction is usually used to provide the most conservative threshold in GWAS. However, due to the linkage imbalance between markers, this threshold line often leads to the generation of false negatives [30]. In this study, we found that the Bonferroni correction was also too strict, and very few significant association sites were found in the sugar beet genome. Therefore, we choose 4.5 as the common threshold for this study. As expected, a comparatively high number of significantly associated SNPs per trait was observed. Through alignment against the sugar beet reference genome, several candidate genes were searched within 25 kb regions of these SNP loci (Tables S3 and S4; Figs. 2, 3, 4, 5, 6 and 7; Figs. S2-S8).

The taproot, which is developed from principal root and hypocotyl tissues [31], is the main sugar storage organ of sugar beet, and its internal and external traits are directly related to the mechanized production of sugar and improvement in sugar content and root biomass yield of sugar beet [32]. In this study, the root traits related to shape, colour, size and texture of RS, RGD, FC and CS were examined, and several putative genes were identified. It has been shown that plant root morphological changes are affected by nutrient elements [33], such as nitrogen (N). For RGD, we found a gene (*BVRB_5g097760*) encoding protein NRT1/PTR FAMILY 6.3 related to the SNW_017567419.1_180782 marker. The NRT/PTR family has been reported to transport nitrate, and many members have been shown to be essential for the development of lateral and primary roots. In a study aiming to identify SNPs linked to the root elongation rate (RER) in sugar beet, the SNP10139 sequence was mapped to the peptide transporter (PTR) gene, a carrier that influences root elongation [34]. In addition, *MdNRT2.1* has a direct role in adventitious root formation and development [35]. Another gene (*BVRB_8g182070*) encoding nudix hydrolase 15 in mitochondria was also associated with RGD. The product of this gene has the capacity to hydrolyse NADPH, which is an essential cofactor required for cell growth and proliferation in the main organs (roots and leaves) [36]. In a study of continuous storage root formation and bulking, a nudix hydrolase homologue was also found in sweet potato using the GWAS approach, and it might be associated with hormones promoting lateral root initiation in young root portions [37, 38]. A particular gene (*BVRB_8g181550*) encoding the protein TRANSPORT INHIBITOR RESPONSE (TIR) 1 was found to be associated with both RGD and RS. It has been proven that Tir2 is required for temperature-dependent hypocotyl elongation. TIR2 expression in the proximal root meristem is auxin sensitive, and root curvature is associated with increased TIR2 expression in the lower epidermal cells and concomitant loss of expression on the upper side [39]. The effect of increasing the asymmetry in auxin concentration amplifies the changes in the root growth response. Close to the markers of SNW_017567326.1_144308, the gene of ethylene-responsive transcription factor ERF014 (*BVRB_1g000090*) was associated with FC. ERFs have been proven to play important roles in stress responses, plant hormones and fruit ripening, including pigment changes. Colour change in fruits due to lycopene accumulation mainly results from the regulation of *LCYb*, which is activated by *CsERF061* in citrus colouration through carotenoid biosynthesis [40]. For CS, the transcription factor MYB77 (*BVRB_2g023500*) exhibited a strong potential association due to its involvement in the auxin response. In MYB77-knockout *Arabidopsis thaliana*, the expression of this auxin-responsive gene was greatly attenuated, and the lateral root density in the MYB77 knockout was lower than that in the wild type under low concentrations of indole-3-acetic acid and under low-nutrient conditions [41]. These effects might be due to the interaction of MYB77 with ARFs to modulate auxin signal transduction and lateral root growth [42]. Auxin plays an essential role in root development.

For sugar beet, the agronomic traits of leaves, hypocotyls, and pollen and other plant morphological traits are also very important, and they are closely related to growth and development and resistance to biotic and abiotic stresses. Sugar beet usually displays the red or green HC phenotype. It has been reported that the *R* locus contains a dominant allele that leads to a red hypocotyl [43]. The gene *BvCYP76AD1*, which represents the *R* locus, encodes a cytochrome P450 enzyme that is required for betalain biosynthesis [44]. However, in this study, we found that two genes, UDP-glycosyltransferase 79B6 (*BVRB_9g223780*) and NAC domain-containing protein 7 (*BVRB_5g097990*), might be associated with beet HC. The former was characterized to be involved in anthocyanin accumulation in *Medicago truncatula* [45]; the latter was proven to participate in carotenoid metabolism in tomato and melon [46, 47]. For PT, a gene encoding the F-box protein At1g10780 (*BVRB_6g140760*) was identified. In *M. truncatula*, this type of protein was found to participate in the processes of indirect somatic embryogenesis and symbiotic nodulation and to be involved in cell division activation and cell cycle control. The transgenic lines exhibited variations in root and hypocotyl growth, leaf and silique development, ploidy level, and leaf parameters [48]. PL might be related to the transport of nutrients such as Pi. A gene (*BVRB_3g048660*) encoding phosphate transporter PHO1 was observed close to the significantly correlated marker SNW_017567376.1_673904. Phosphorus can promote plant photosynthesis, promote root development, and make the stem stronger, which is beneficial to the early growth of seedlings. PHO1 is a Pi efflux transporter responsible for xylem loading of Pi in organs such as roots [49]. As sugar beet is a self-incompatible hybrid crop, PQ is very important for its seed production. Three genes probably associated with PQ were identified. *BVRB_5g106130* encodes AP-2 complex subunit mu, which is a medium subunit of the heterotetramer AP2. The *A. thaliana* mutant *ap2m* displays multiple defects in pollen production and viability and in elongation of staminal filaments and pollen tubes, all of which are pivotal processes needed for fertilization [50]. *BVRB_2g041790* is annotated as the trihelix transcription factor ASIL2. There are two transitions involved in the induction of the embryo maturation programme midway through seed development and its repression during the vegetative phase of plant growth. The trihelix transcription factors of *Arabidopsis* ASIL1 and ASIL2 have been proposed to repress maturation both embryonically and postembryonically [51, 52]. Late embryogenesis abundant proteins were originally discovered in the late stages of embryo development in cotton seeds [53]. Pollen is known to undergo programmed desiccation during development, as does seed maturation, and a novel pollen-specific LEA-like protein (LP28) in *Lilium longiflorum* was abundant in cytoplasmic granules of the vegetative cell until pollen maturation, but after hydration, it appeared in the elongating pollen tube wall [54]. In this study, *BVRB_5g106150* was found to associate with PQ and encode late embryogenesis abundant protein 18, which might influence pollen maturation. Sugar beet has no apparent epicotyl, and the basal leaves are clustered, with long petioles. The FLT mainly presents as semicrawling, which is very conducive to the absorption of light and the improvement in sugar beet photosynthetic efficiency. The transcription factor MYB77 (*BVRB_2g023500*) and alcohol acyltransferase 9 (*BVRB_2g023460*) might be involved in basal leaf clustering because of their putative role in the auxin response or ester biosynthesis [55, 56]. The size of leaf organs is determined by the interplay of cell proliferation and expansion, and some F-box proteins participate in plant organ morphogenesis [57]. In this study, we also found that the F-box protein CPR1 (*BVRB_8g181140*), close to two markers, SNW_017567490.1_421148 and SNW_017567490.1_421085, was associated with C. Its expression might influence the cell division rate during the early stages of leaf development, similar to the F-box protein AtFBX92 [58].

Nevertheless, there were still some traits that either could not be associated with candidate genes (SR) or were associated with genes whose function seemed to have no putative relationship with the traits themselves (GV and PW). The materials that we selected had high diversity, rich variation, and many SNP variations. During sequence alignment, such variants may be filtered out due to the low mapping rate, resulting in the loss of SNPs. These findings may also be due to the large difference in the quantity of these traits, which leads to the filtering out of variation as noise during the screening process.

## Conclusion

Here, we associated phenotypes (13 descriptive agronomic traits) and genotypes using 170,750 GBS-derived SNPs after precise evaluation of the population structure and genetic diversity of 977 sugar beet germplasms. Through GWASs, several candidate genes linked with 159 significant SNPs were identified, and a number of interesting genes were inferred to be functional in the morphological variation of taproots and the growth and development of sugar beet. Future sugar beet breeding efforts must make use of the genetic and genomic resources available for efficient improvement.

## Materials and methods

### Plant materials and morphological assessment

The 977 studied sugar beet germplasms came from 21 countries (Table S1) and are preserved in the National Beet Medium-term Gene Bank (https://www.cgris.net/query/croplist.php). The experiment was carried out in the Hulan Experimental field (latitude 45.997°N, longitude 126.628°E) of Heilongjiang University in 2015–2018. The field design followed a randomized block arrangement, with two rows of blocks and three repetitions. Each row is 10 m long and planted with approximately 55 sugar beets. Three sugar beet germplasms with stable agronomic traits were used as annual experimental controls. Thirteen agronomic, growth and development components and quality-related traits were evaluated, including pollen quantity (PQ), plant type (PT), hypocotyl colour (HC), cotyledon size (C), petiole width (PW), petiole length (PL), fascicled leaf type (FLT), root shape (RS), crown size (CS), root groove depth (RGD), skin roughness (SR), flesh colour (FC) and growth vigour (GV). These descriptive traits were investigated and recorded under field growth conditions and were assigned and defined according to “the Descriptors and Date Standard for Beet (*Beta vulgaris* L.)” [26, 59].

In each experimental plot, PQ is the amount of pollen in the stamens of sugar beets during flowering. “Little” is described as “anthers are light yellow, with few pollen scattered after the anthers open”; The “medium” amount of pollen shows relatively yellow anthers, after which crack, a considerable portion of the pollen is scattered; “Much” means that the anthers are very yellow, and after they crack, a large amount of mature pollen is scattered. PT is described as the phenotype of the main stems and lateral branches of > 70% of sugar beets during their blooming stage. HC is the colour of smooth parts below the cotyledons of > 90% of sugar beet seedlings, and in terms of the cotyledon area, the cotyledon size (C) is divided into small (< 99.8 mm^2^), medium (≥ 99.8 mm^2^, < 126.5 mm^2^), and large (≥ 126.5 mm^2^). In the flourishing vegetative growth stage of sugar beet, PW is described as the width of the thickest part of the petiole in the middle layer of the plant’s leaf cluster, and 0.8 and 1.3 cm are the critical values defining narrow, medium and broad; PL is the length from the base of the petiole to the base of the longest leaf, where short, medium and long is classified as < 20 cm, ≥ 20 and < 32 cm, and ≥ 32 cm, respectively; the erect, semicrawl, and crawl types (FLT) are described according the angles (70° and 30°) between most of the plant’s petioles and the ground. During the sugar beet harvest period, we observed the external shape of the taproot (RS, 50%), the depth of the root grooves (RGD) on both sides of the taproot, the smoothness of the taproot surface (SR), and the colour of the flesh inside the taproot skin (SR); the size of the crown taproot (CS, the proportion of the upper part of the taproot to the entire taproot) was defined as small (< 10%), medium (≥ 10%, < 20%) and large (≥ 20%). The seedling growth vigour (GV) was divided into five levels and defined as the strength and degree of vigour of seedling growth after emergence and before seedling setting.

The phenotypic data were analysed using Microsoft Excel 2010, and the mean value, standard deviation and CV were calculated according to Götze et al. [60]. The Shannon–Weiner index of genetic diversity (H’) was used to analyse the genetic diversity of the descriptive traits. The formula was as follows:

1 $${H'}=-\sum _{i=1}^{n}{P}_{i}Ln{P}_{i}$$

where *P*_i_ represents the percentage of the number of materials with the ith character relative to the total number. *Ln* is the natural logarithm.

### Genotyping-by-sequencing and data analyses

A total of 977 accessions of sugar beet were genotyped using a GBS approach. Fresh leaf tissue was harvested from the seedlings and stored at -80 °C. The purified and integrated genomic DNA was quantified and digested with restriction endonuclease. Each sample was amplified after adding a connector with a barcode and was used to construct the GBS library. Then, sequencing was performed using the Illumina HiSeq PE150 platform. The original image data obtained by high-throughput sequencing were converted into raw data through base calling. After strict filtering of sequencing data, such as reads containing the connector sequence, paired reads with an N content exceeding 10% or low-quality (≤ 5) reads exceeding 50% in single-end sequencing, high-quality clean data were obtained. Then, the effective high-quality sequencing data were compared to the sugar beet reference genome (https://ftp.ncbi.nlm.nih.gov/genomes/all/GCF/000/511/025/GCF_000511025.2_RefBeet-1.2.2/GCF_000511025.2_RefBeet-1.2.2_genomic.fna.gz) using Burrows‒Wheeler Alignment (BWA) software (parameter: mem-t 4-k 32-M; 0.7.17).

SAMtools (1.9) was used to transform the format of the .sam file and build an index to generate a .bai file. Genome Analysis Toolkit (GATK; 4.2.6.1) and ANNOVAR [61] were used for SNP detection and population SNP annotation, respectively. After filtering under the conditions of DP ≥ 4, missing data ≤ 80% and minor allele frequency (MAF) ≥ 0.05, high-quality SNPs were obtained for subsequent analysis.

### Population hierarchical analysis, kinship and LD decay

Pairwise genetic distances among the 977 beet accessions were calculated. Phylogenetic clustering was performed and displayed by EvolView (www.evolgenius.info/evolview) using the NJ method (Fig. 1a and b). VCFtools (0.1.17) was used to convert the .vcf files to .ped files, and then PLINK (1.9) software was used to convert from .ped format to .bed format. To assess genetic structure (.bed files), the Admixture-based clustering model was applied using Admixture (1.3.0) software. The population size K value ranged from 1 to 9, representing the simulated number of groups in ancient populations. The optimal K was chosen to determine the optimal number of classifications using Admixture 1.3.0, and the results were visualized in R (4.1.0) software (Fig. 1e and f). PCA of high-quality SNPs was performed using Tassel (5.2.82) software. The eigenvector decomposition of the matrix was performed in R. The first two principal components (PCs) were plotted and visualized using R (4.1.0) software, and Fig. 1c was drawn using the ggplot2 package of R software. Estimation of LD in the 977 sugar beet germplasms was performed between SNPs on each chromosome, and it was estimated based on *r*^*2*^ using PopLDdecay (3.4.2), and ggplot2 package was also used to produce Fig. 3. The kinship (K) analysis was performed using Tassel (5.2.82) software to obtain the kinship matrix reflecting the relatedness among individuals, and the results were visualized using the pheatmap package in R (4.1.0) (Fig. 1d).

### Genome-wide association analyses

The GWAS technique was used to carry out phenotype–genotype association analysis of the 13 phenotypic traits in the sugar beet population. GWAS was conducted on the datasets of SNPs and these observed descriptive traits. An MLM was generated by Tassel (5.2.82) software to determine the associations using the incorporated PCA and kinship results from population structure analysis (the random effect based on the genetic relatedness across all accessions). Population structure and kinship can effectively reduce false-positive results in mixed models.

Bonferroni corrected significance threshold (-log_10_ (0.05/total SNPs) ≈ 6.53) was used as the standard cutoff in GWAS analysis. Due to its excessive conservatism and strictness, there were very few markers associated *p*-values in sugar beet genome that can meet this standard. Thus, we adjusted the threshold to 4.5 according to our and others’ experience [30]. According to the physical location and *P* value of these high-quality variation sites in the beet genome, Manhattan (Fig. 2) and quantile‒quantile (Q-Q) (Fig. S1) plots were drawn in combination based on genotype–phenotype associations with the CMplot package of R (4.0.1). After screening for false-positive SNPs according to genome annotation, we obtained high-quality and significant SNPs with a threshold of -log_10_*p* ≥ 4.5.

### Candidate gene identification

The genomic regions within the LD block of the significantly correlated SNPs meeting the threshold of -log_10_*p* ≥ 4.5 were selected to identify candidate genes and haplotype analysis (Figs. 4, 5 and 6; Figs. S2-S8), and the results were visualized by LD block show software [62]. Putative candidate genes were proposed for each locus using the gene annotation databases of NCBI (http://www.ncbi.nlm.nih.gov/) and UniProt (https://www.uniprot.org/). The target trait related genes and chromosome data were inputted into the online analysis tool GENESCLOUD, and the chord graph drawing function (https://www.genescloud.cn/chart/ActiveChordPlot) was used to visualize the relationship between them (Fig. 7).

## Electronic supplementary material

Below is the link to the electronic supplementary material.

**Additional file 1**: **Table S1** Basic information of 977 sugar beet germplasms and their SNPs identified using the GBS approach; **Table S2** The results of SNP statistics and annotation; **Table S3** List of SNPs significantly associated with 13 descriptive traits in 977 sugar beet accessions by GWAS; **Table S4** Candidate genes located in the sweep region.

**Additional file 2**: **Fig. S1** Quantile-quantile (QQ) plots of 13 descriptive traits constructed using a mixed linear model (MLM). PQ, pollen quantity; PT, plant type; HC, hypocotyl colour; C, cotyledon size; PW, petiole width; PL, petiole length; FLT, fascicled leaf type; RS, root shape; CS, crown size; RGD, root groove depth; SR, skin roughness; F, flesh colour; GV, growth vigour. **Fig. S2** Manhattan plot and LD heatmap of the candidate genes for RS. The orange vertical line indicates the position of the associated SNPs, and the orange horizontal line indicates -log_10_*p*. **Fig. S3** Manhattan plot and LD heatmap of the candidate genes for C. The orange vertical line indicates the position of the associated SNPs, and the orange horizontal line indicates -log_10_*p*. **Fig. S4** Manhattan plot and LD heatmap of the candidate genes for PQ. The orange vertical line indicates the position of the associated SNPs, and the orange horizontal line indicates -log_10_*p*. **Fig. S5** Manhattan plot and LD heatmap of the candidate genes for CS. The orange vertical line indicates the position of the associated SNPs, and the orange horizontal line indicates -log_10_*p*. **Fig. S6** Manhattan plot and LD heatmap of the candidate genes for FC. The orange vertical line indicates the position of the associated SNPs, and the orange horizontal line indicates -log_10_*p*. **Fig. S7** Manhattan plot and LD heatmap of the candidate genes for PT. The orange vertical line indicates the position of the associated SNPs, and the orange horizontal line indicates -log_10_*p*. **Fig. S8** Manhattan plot and LD heatmap of the candidate genes for PL. The orange vertical line indicates the position of the associated SNPs, and the orange horizontal line indicates -log_10_*p*.

## Data Availability

The original datasets analysed in the current study are available on the SRA database under Bioproject accession PRJNA948801 (https://www.ncbi.nlm.nih.gov/bioproject/PRJNA948801).

## Abbreviations

C: Cotyledon size
CS: Crown size
CV: Coefficient of variation
FC: Flesh colour
FLT: Fascicled leaf type
GBS: Genotyping-by-sequencing
GV: Growth vigour
GWAS: Genome-wide association study
HC: Hypocotyl colour
LD: Linkage disequilibrium
MLM: Mixed linear model
NJ: Neighbour-joining
PCA: Principal component analysis
PL: Petiole length
PQ: Pollen quantity
PT: Plant type
PW: Petiole width
Q–Q: Quantile–quantile
RGD: Root groove depth
RS: Root shape
SNP: Single-nucleotide polymorphism
SR: Skin roughness
