# Supplementary material for: Genetic diversity and genome-wide association study of 13 agronomic traits in 977 Beta vulgaris L. germplasms
Source: BMC Genomics. 2023 Jul 24;24:413. doi: 10.1186/s12864-023-09522-y (PMC10364417; doi:10.1186/s12864-023-09522-y)
Supplement: Supplementary file 2 — Additional file 2: Fig. S1 Quantile-quantile (QQ) plots of 13 descriptive traits constructed using a mixed linear model (MLM). PQ, pollen quantity; PT, plant type; HC, hypocotyl colour; C, cotyledon size; PW, petiole width; PL, petiole length; FLT, fascicled leaf type; RS, root shape; CS, crown size; RGD, root groove depth; SR, skin roughness; F, flesh colour; GV, growth vigour. Fig. S2 Manhattan plot and LD heatmap of the candidate genes for RS. The orange vertical line indicates the position of the associated SNPs, and the orange horizontal line indicates -log10p. Fig. S3 Manhattan plot and LD heatmap of the candidate genes for C. The orange vertical line indicates the position of the associated SNPs, and the orange horizontal line indicates -log10p. Fig. S4 Manhattan plot and LD heatmap of the candidate genes for PQ. The orange vertical line indicates the position of the associated SNPs, and the orange horizontal line indicates -log10p. Fig. S5 Manhattan plot and LD heatmap of the candidate genes for CS. The orange vertical line indicates the position of the associated SNPs, and the orange horizontal line indicates -log10p. Fig. S6 Manhattan plot and LD heatmap of the candidate genes for FC. The orange vertical line indicates the position of the associated SNPs, and the orange horizontal line indicates -log10p. Fig. S7 Manhattan plot and LD heatmap of the candidate genes for PT. The orange vertical line indicates the position of the associated SNPs, and the orange horizontal line indicates -log10p. Fig. S8 Manhattan plot and LD heatmap of the candidate genes for PL. The orange vertical line indicates the position of the associated SNPs, and the orange horizontal line indicates -log10p. [file 12864_2023_9522_MOESM2_ESM.docx]

**Genetic diversity and genome-wide association study of 13 agronomic traits in 977 *Beta vulgaris* L. germplasms**

Dali Liu^1,2^, Wenbo Tan^1,2^, Hao Wang^1,2^, Wangsheng Li^1,2^, Jingjing Fu^1,2^, Jiajia Li^1,2^, Yuanhang Zhou^3^, Ming Lin^3^, Wang Xing^1,2,*^

1 National Beet Medium-term Gene Bank, Heilongjiang University, Harbin, 150080, P. R. China

2 Key Laboratory of Sugar Beet Genetics and Breeding, College of Advanced Agriculture and Ecological Environment, Heilongjiang University, Harbin 150080, P. R. China

3 Xinjiang Academy of Agricultural Sciences, Urumqi 830091, P. R. China

* Correspondence: xyjiayou_086@163.com; Tel.: +86 451 86609494

**Supplementary Figures**


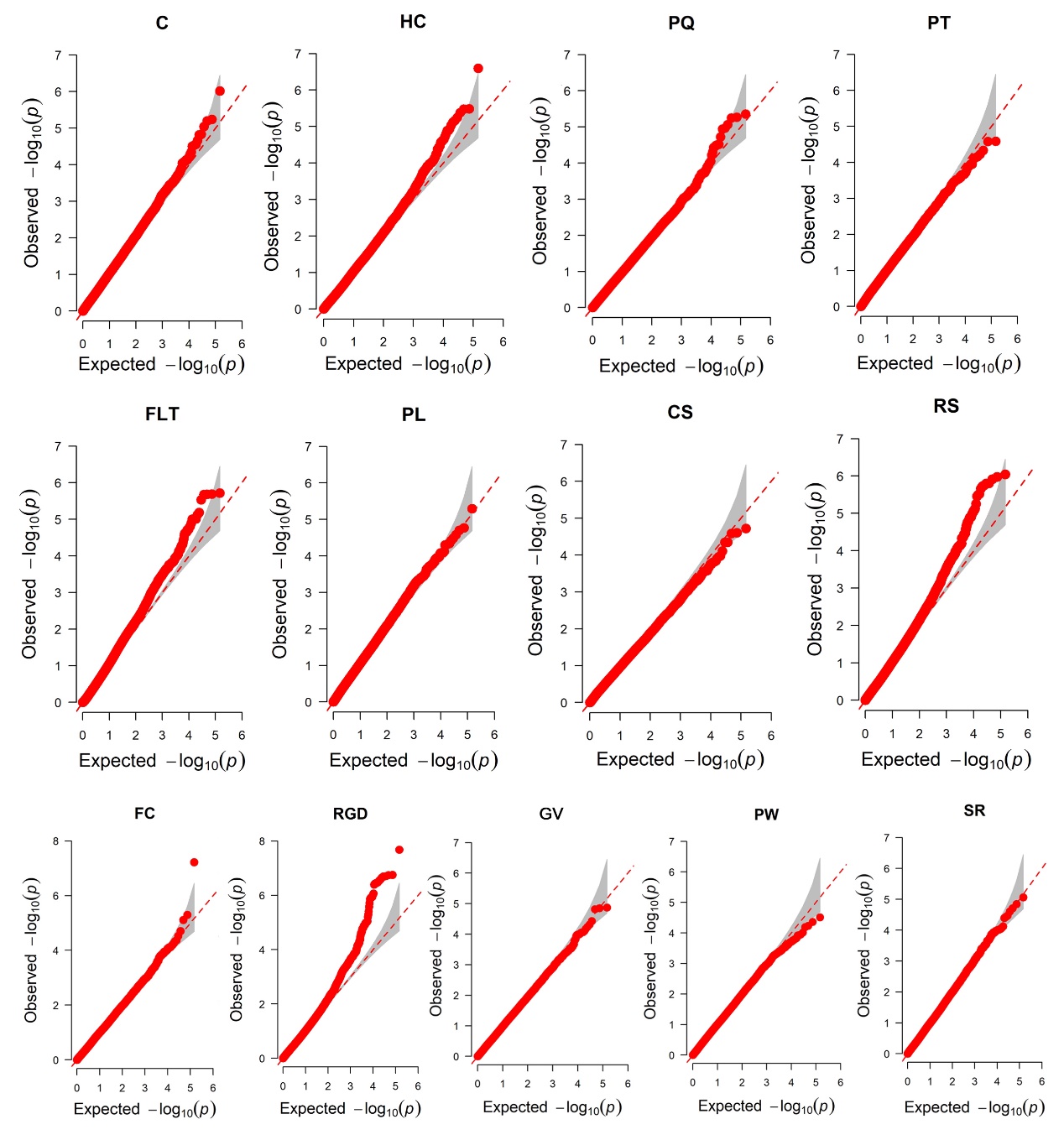


**Fig. S1** Quantile–quantile (QQ) plots of 13 descriptive traits constructed using a mixed linear model (MLM). PQ, pollen quantity; PT, plant type; HC, hypocotyl colour; C, cotyledon size; PW, petiole width; PL, petiole length; FLT, fascicled leaf type; RS, root shape; CS, crown size; RGD, root groove depth; SR, skin roughness; F, flesh colour; GV, growth vigour.


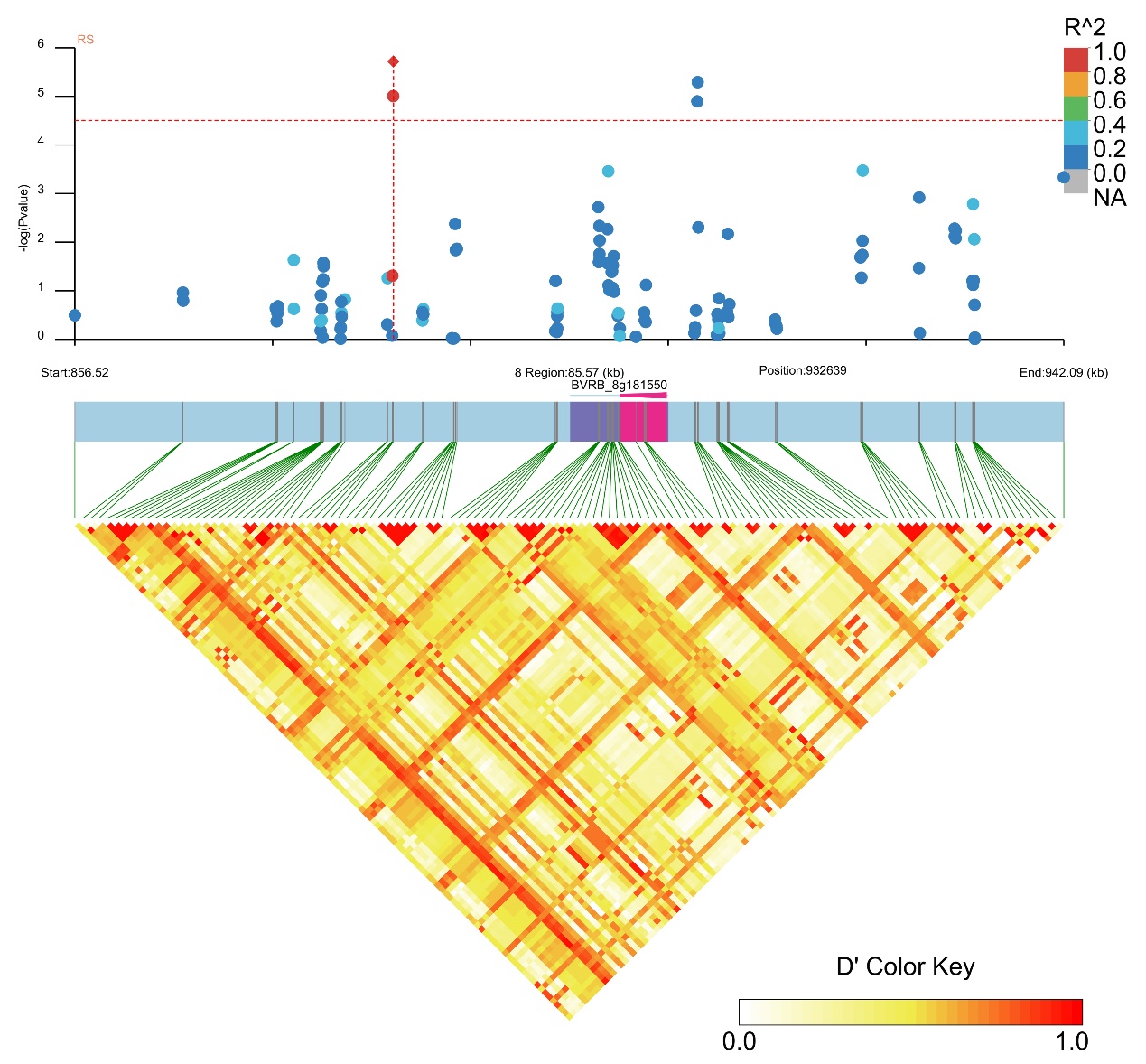


**Fig. S2** Manhattan plot and LD heatmap of the candidate genes for RS. The orange vertical line indicates the position of the associated SNPs, and the orange horizontal line indicates -log_10_p.


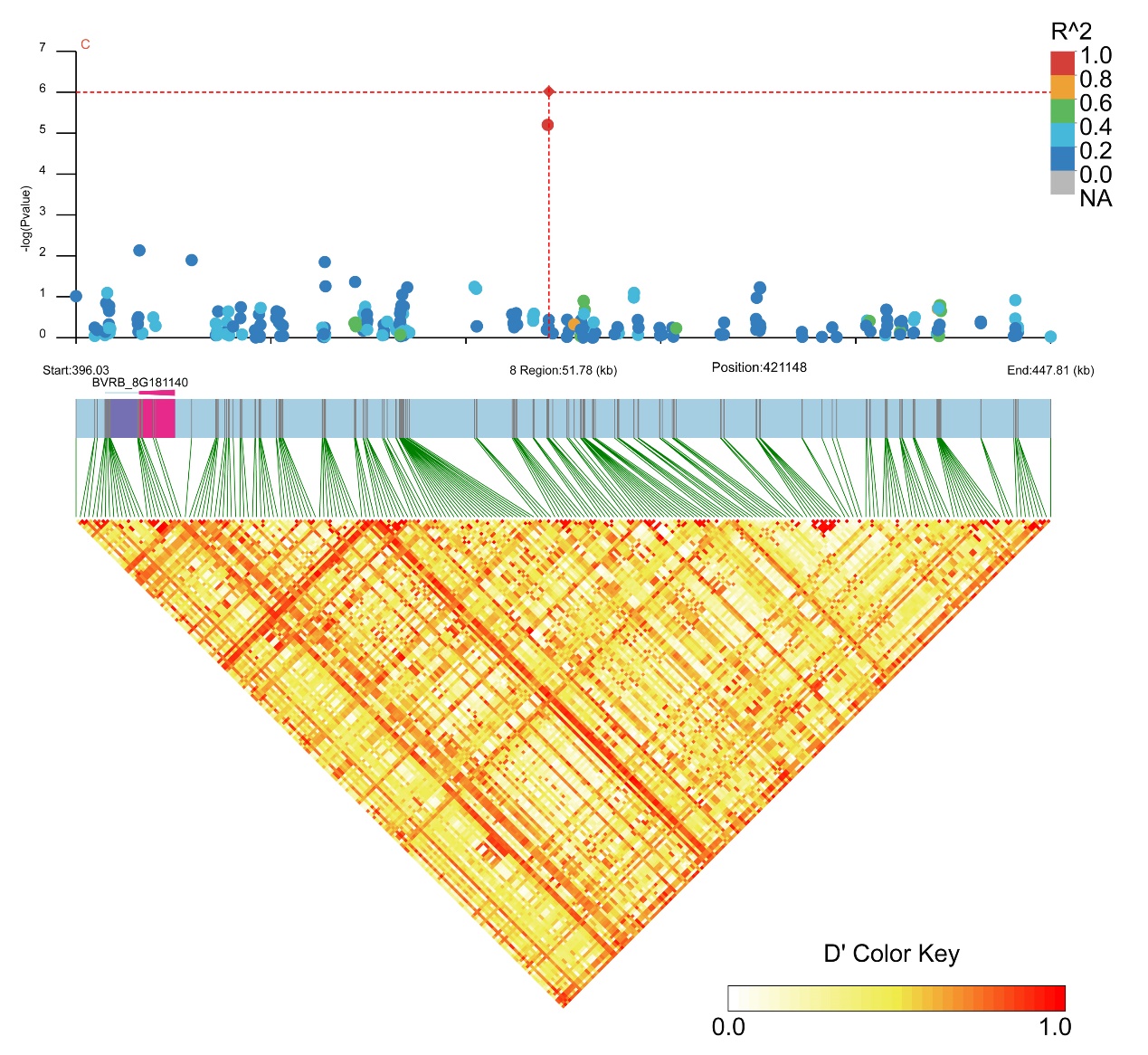


**Fig. S3** Manhattan plot and LD heatmap of the candidate genes for C. The orange vertical line indicates the position of the associated SNPs, and the orange horizontal line indicates -log_10_p.


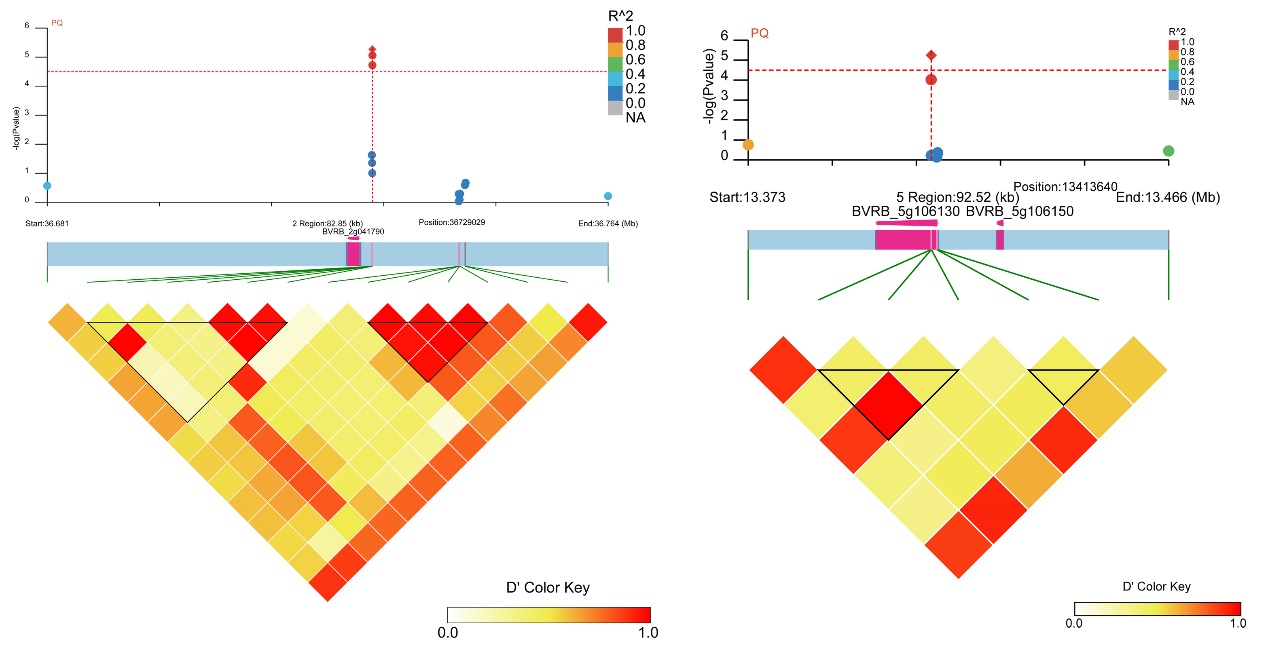


**Fig. S4** Manhattan plot and LD heatmap of the candidate genes for PQ. The orange vertical line indicates the position of the associated SNPs, and the orange horizontal line indicates -log_10_p.


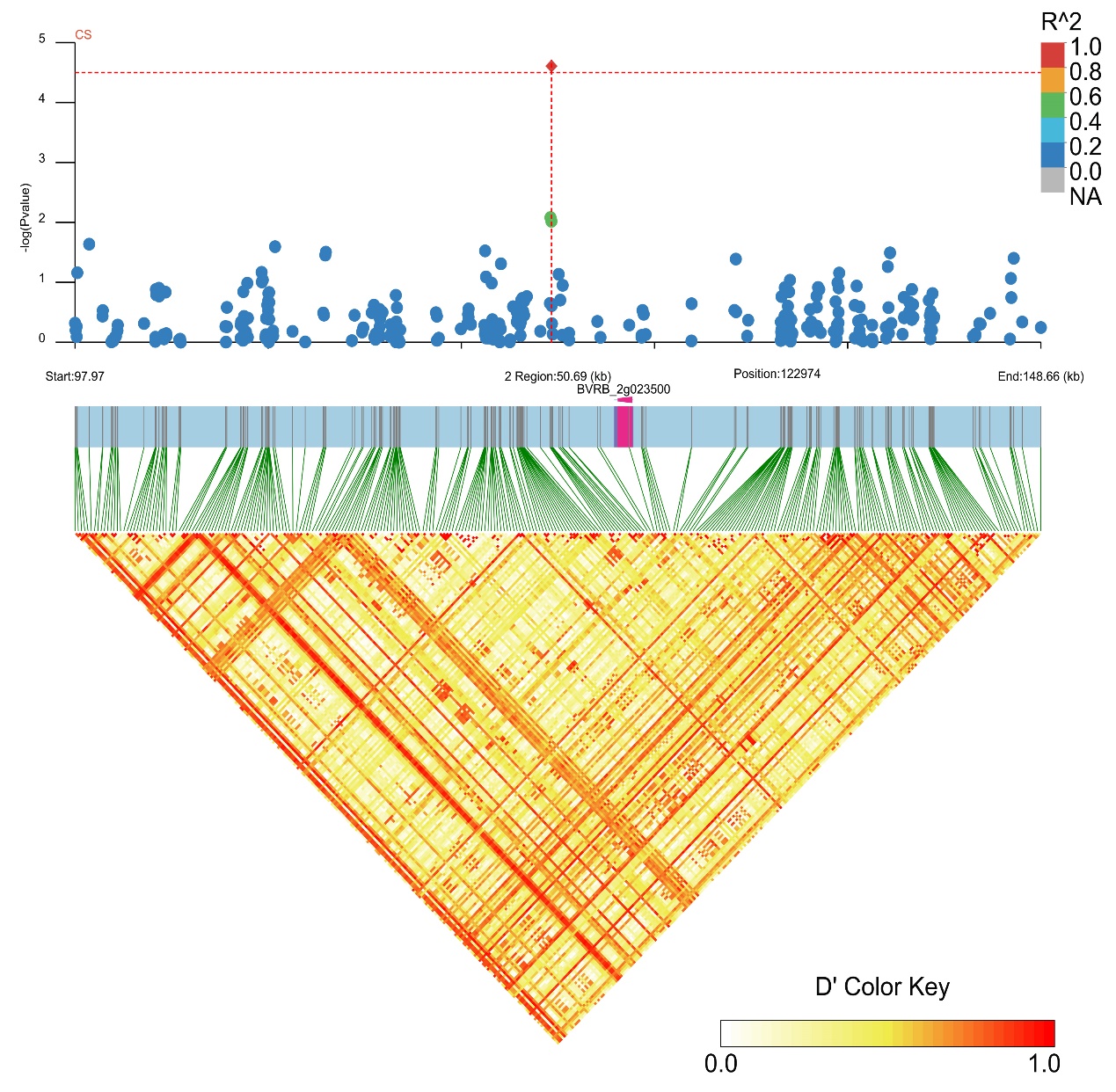


**Fig. S5** Manhattan plot and LD heatmap of the candidate genes for CS. The orange vertical line indicates the position of the associated SNPs, and the orange horizontal line indicates -log_10_p.


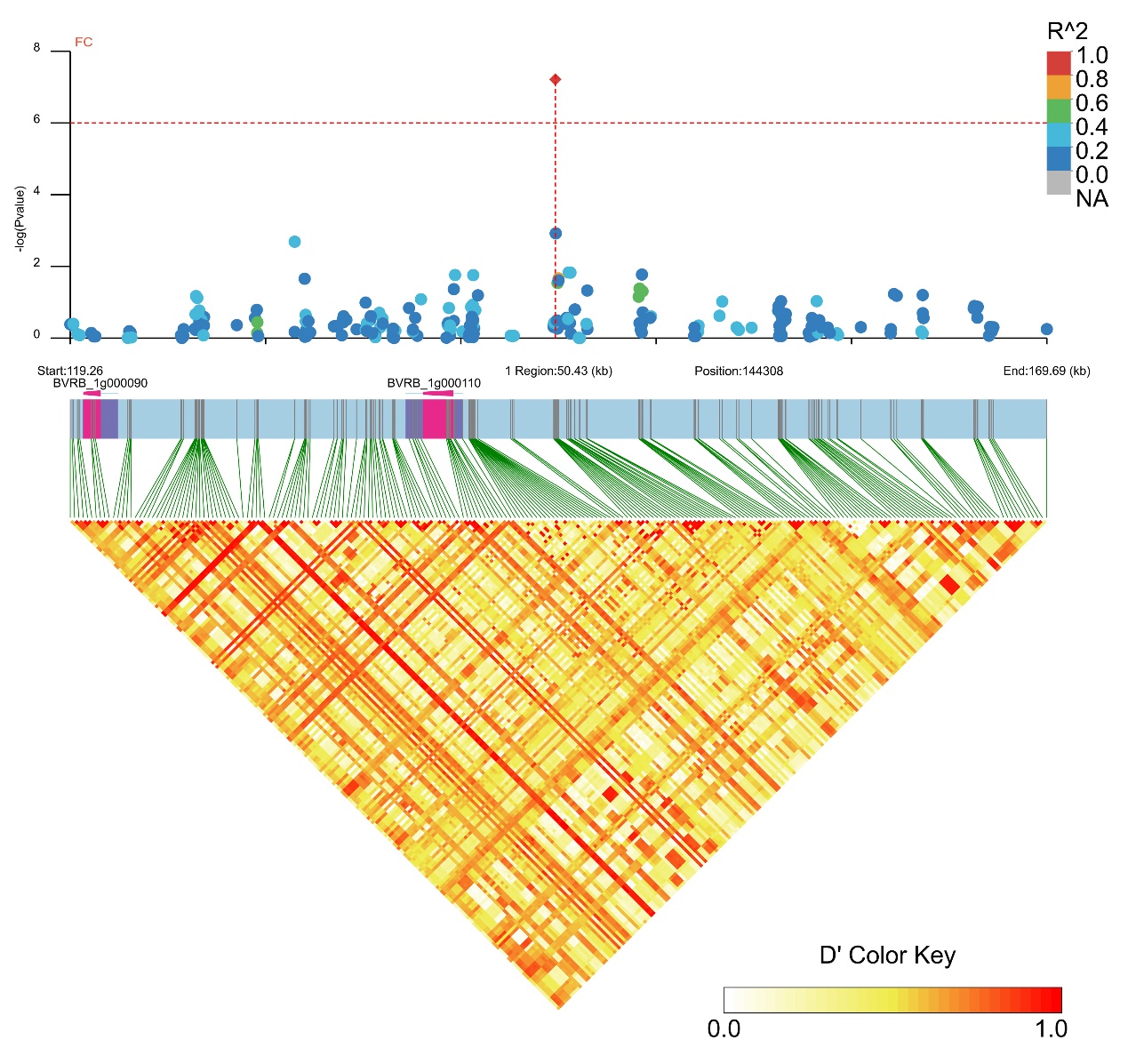


**Fig. S6** Manhattan plot and LD heatmap of the candidate genes for FC. The orange vertical line indicates the position of the associated SNPs, and the orange horizontal line indicates -log_10_p.


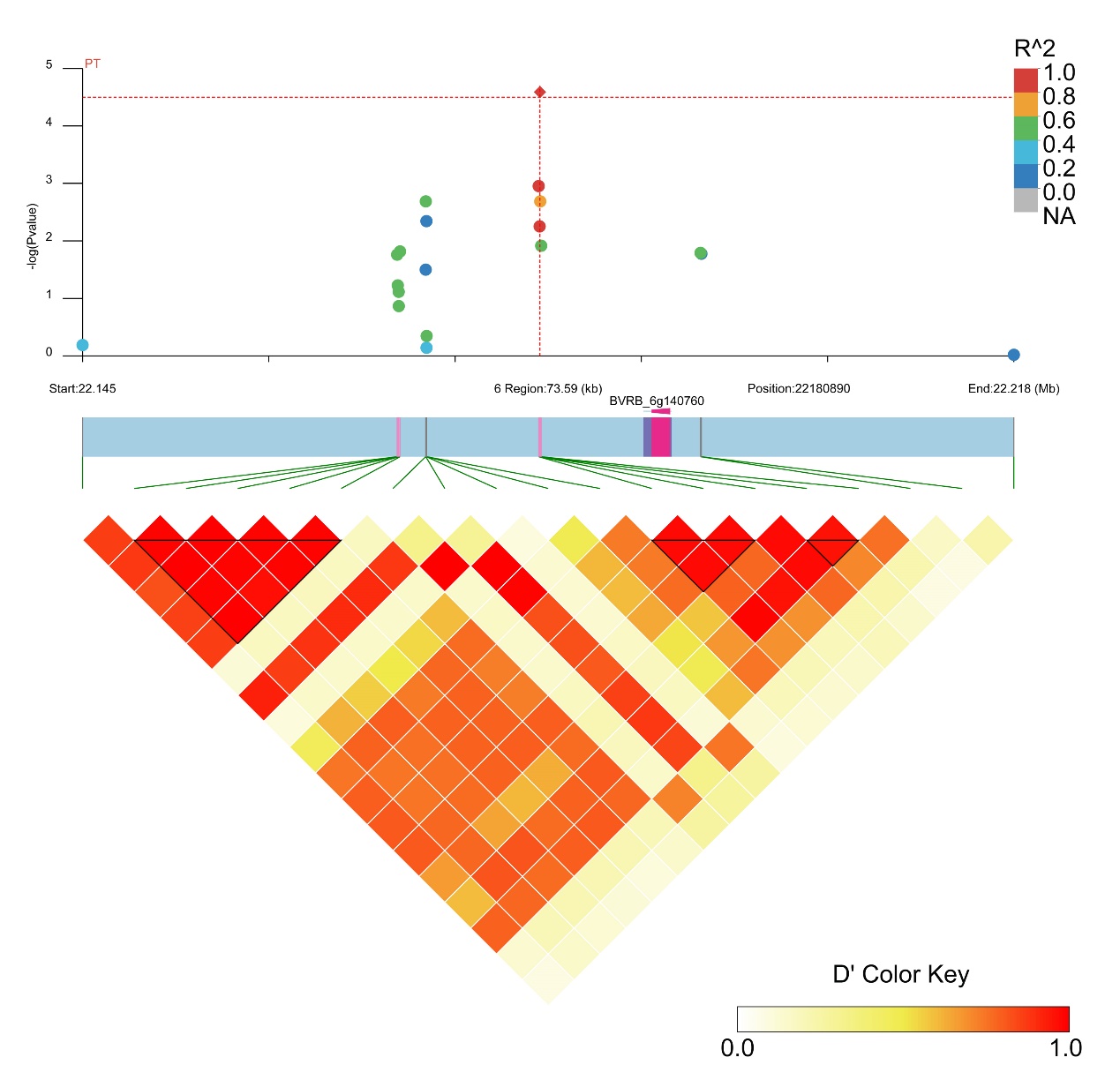


**Fig. S7** Manhattan plot and LD heatmap of the candidate genes for PT. The orange vertical line indicates the position of the associated SNPs, and the orange horizontal line indicates -log_10_p.


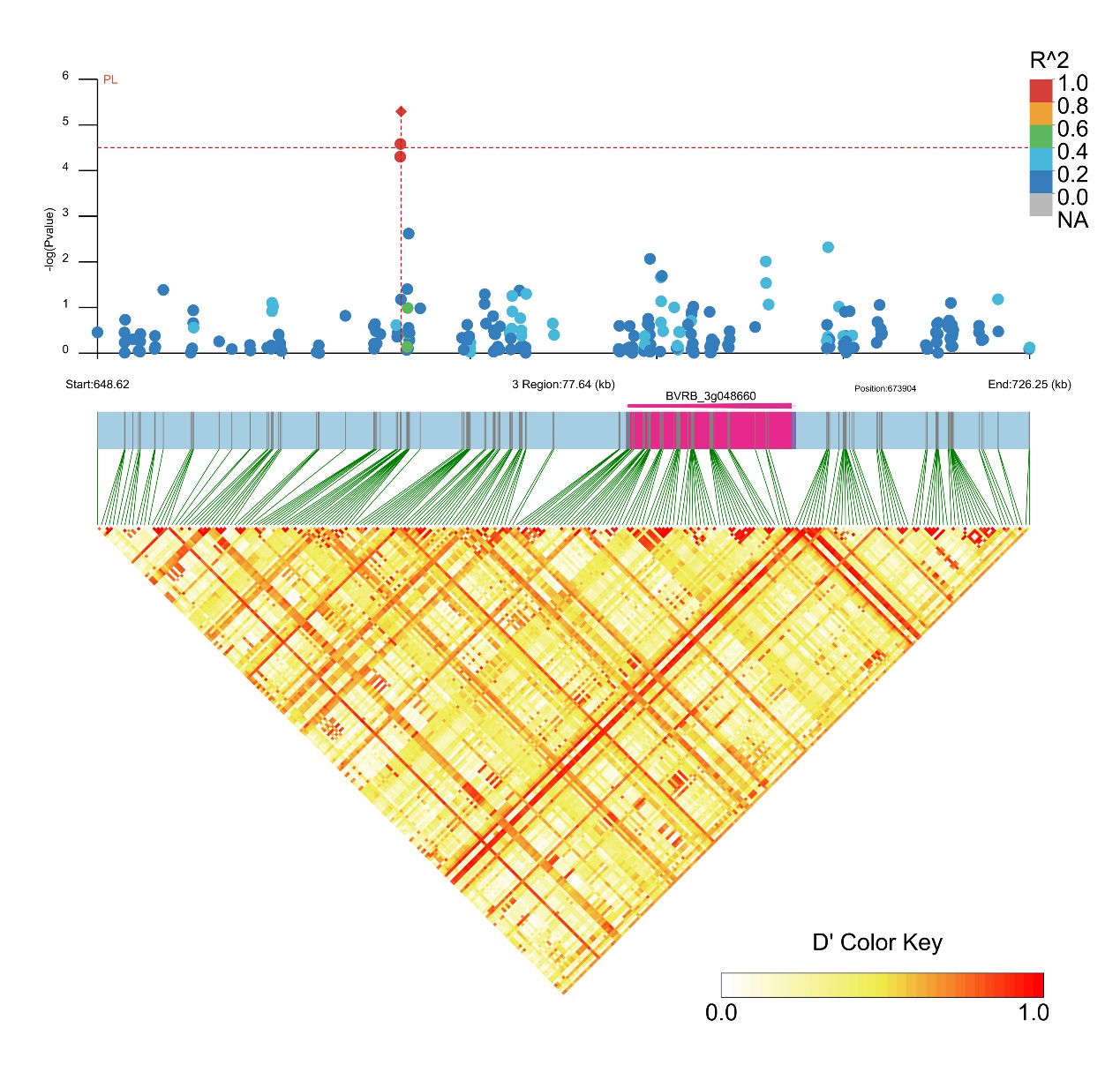


**Fig. S8** Manhattan plot and LD heatmap of the candidate genes for PL. The orange vertical line indicates the position of the associated SNPs, and the orange horizontal line indicates -log_10_p.
